# Hypoglycemia is associated with a higher risk of mortality and arrhythmias in ST-elevation myocardial infarction, irrespective of diabetes

**DOI:** 10.3389/fcvm.2022.940035

**Published:** 2022-10-10

**Authors:** Basel Humos, Ziyad Mahfoud, Soha Dargham, Jassim Al Suwaidi, Hani Jneid, Charbel Abi Khalil

**Affiliations:** ^1^Department of Research, Weill Cornell Medicine-Qatar, Doha, Qatar; ^2^Heart Hospital, Hamad Medical Corporation, Doha, Qatar; ^3^The Michael E. DeBakey VA Medical Centre, Baylor College of Medicine, Houston, TX, United States; ^4^Joan and Sanford I. Weill Department of Medicine, Weill Cornell Medicine, New York, NY, United States

**Keywords:** myocardial infarction, hypoglycemia, diabetes, mortality, arrhythmias, cardiovascular disease, ST-elevation myocardial infarction

## Abstract

**Aims:**

We aimed to assess the impact of hypoglycemia in ST-elevation myocardial infarction (STEMI).

**Background:**

Hypoglycemia increases the risk of mortality in patients with diabetes and high cardiovascular risk.

**Methods:**

We used the National Inpatient Sample (2005–2017) database to identify adult patients with STEMI as the primary diagnosis. The secondary diagnosis was hypoglycemia. We compared cardiovascular and socio-economic outcomes between STEMI patients with and without hypoglycemia and assessed temporal trends.

**Results:**

Hypoglycemia tends to complicate 0.17% of all cases hospitalized for STEMI. The mean age (±SD) of STEMI patients hospitalized with hypoglycemia decreased from 67 ± 15 in 2005 to 63 ± 12 in 2017 (*p* = 0.046). Mortality was stable with time, but the prevalence of ventricular tachycardia, ventricular fibrillation, acute renal failure, cardiogenic shock, total charges, and length of stay (LOS) increased with time (*p* < 0.05 for all). Compared to non-hypoglycemic patients, those who developed hypoglycemia were older and more likely to be black; only 6.7% had diabetes compared to 28.5% of STEMI patients (*p* = 0.001). Cardiovascular events were more likely to occur in hypoglycemia: mortality risk increased by almost 2.5-fold (adjusted OR = 2.625 [2.095–3.289]). There was a higher incidence of cardiogenic shock (adjusted OR = 1.718 [1.387–2.127]), atrial fibrillation (adjusted OR = 1.284 [1.025–1.607]), ventricular fibrillation (adjusted OR = 1.799 [1.406–2.301]), and acute renal failure (adjusted OR = 2.355 [1.902–2.917]). Patients who developed hypoglycemia were less likely to have PCI (OR = 0.596 [0.491–0.722]) but more likely to have CABG (OR = 1.792 [1.391–2.308]). They also had a longer in-hospital stay and higher charges/stay.

**Conclusion:**

Hypoglycemia is a rare event in patients hospitalized with STEMI. However, it was found to have higher odds of mortality, arrhythmias, and other comorbidities, irrespective of diabetes.

## Introduction

Despite all the advances in the management of coronary heart disease (CHD) in general and reperfusion therapy in particular, ST-elevation myocardial infarction (STEMI) still represents a significant medical, social, and economic burden worldwide (1). This burden is governed by many predisposing modifiable and non-modifiable risk factors for cardiovascular disease (CVD). For instance, in patients with first myocardial infarction, in-hospital mortality ranges between 3.6 and 14.9% and varies according to the number of risk factors (2).

Glycemic control is a significant risk factor for CVD. In patients with pre-diabetes or diabetes, poor control is associated with an excess of cardiovascular events, including STEMI (3). However, better control is associated with fewer in-hospital and 2-year major cardiovascular events in STEMI patients undergoing primary coronary intervention (4, 5).

While severe hyperglycemia encountered in diabetic ketoacidosis or hyperglycemic hyperosmolar states increases mortality and morbidity in STEMI, the impact of hypoglycemia is unknown. We, therefore, assessed the impact of hypoglycemia in patients hospitalized for STEMI and examined its trend.

## Materials and methods

### Study database

Data from the National Inpatient Sample (6) was analyzed. The NIS is the largest North American public inpatient database in healthcare, developed by the Healthcare Cost and Utilization Project (HCUP) and financed by the Agency for Healthcare Research and Quality (AHRQ) (NIS). NIS collects discharge level data from roughly 1,000 hospitals in the United States, with over 7 million hospital admissions added each year. Discharge weights are used to determine national estimates per the HCUP recommendation (NIS). The weighted data amounts to over 35 million hospital records representing a large national sample. NIS has no identifiable patient information, and all medical diagnoses and interventions are coded using the international classification of disease, 9th version (ICD-9) up till 2014, and the 10th version (ICD-10) 10 codes afterward. The codes have been validated and used in similar NIS studies (7–9) (Supplementary Table 1).

### Study population and outcomes

Patients ≥18-year-old with a primary discharge diagnosis of STEMI between 2005 and 2017 were included in our study. The secondary diagnosis was in-hospital hypoglycemia. Exclusion criteria were unknown age, gender, length of stay, in-hospital outcome, and hospital cost. The primary outcome was in-hospital mortality. Secondary outcomes included: in-hospital ventricular tachycardia, ventricular fibrillation, atrial fibrillation, acute heart failure, stroke, acute renal failure, and procedural outcomes: primary coronary intervention (PCI), thrombolysis, and coronary artery bypass grafts (CABG).

### Statistical analysis

Descriptive statistics were presented as frequencies distributions with percentages for categorical variables, while mean (standard deviation) or medians (interquartile range). Temporal changes were assessed using a trend test based on generalized linear models. A comparison of baseline characteristics of STEMI patients with *vs.* without hypoglycemia was performed using a Student’s *t*-test or a χ^2^ test. We first assessed temporal trends in characteristics and cardiovascular outcomes for every group, then combined data for all years to compare both groups. Multivariable logistic regression analysis was performed to look for predictors of mortality in patients with STEMI and diabetes; an interaction test was also used to look for an association between diabetes and hypoglycemia. Cardiovascular events were adjusted for baseline characteristics that are different among both groups, including age, gender, obesity, hypertension, dyslipidemia, peripheral vascular disease, diabetes, coronary artery disease, renal failure, hospital region, and the primary expected payer. We further used the receiver operating characteristic (ROC) curve to assess the predictive ability of our statistical model for the different outcomes after adjustments (10). Costs were corrected for inflation using rates provided by the US Bureau of labor statistics. A *p*-value <0.05 was considered statistically significant. Analyses were performed using SPSS (IBM, version 22.0) and STATA (version 15).

## Results

**FIGURE 1:**
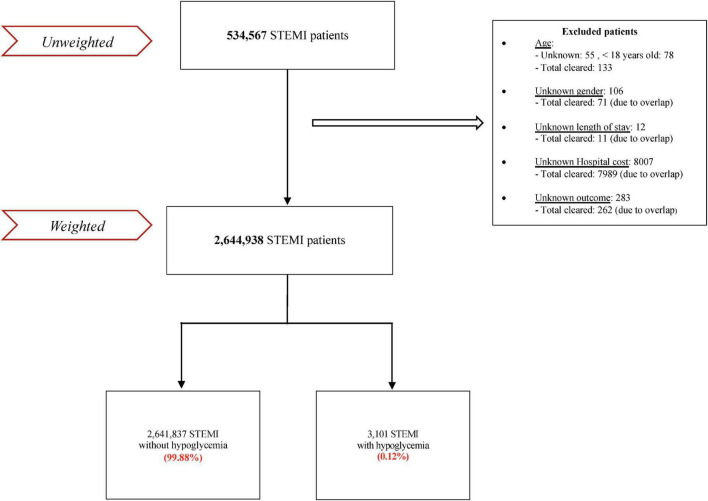
Flowchart of the analysis.

Five hundred thirty-four thousand five hundred sixty-seven patients were admitted for STEMI between January 1, 2005, and December 31, 2017. After excluding patients with missing data (8,466) and subsequently weighting, the total number of patients was 2,644,938, of which 3,101 had hypoglycemia (0.17%) (Figure 1).

### STEMI patients with hypoglycemia

The number of patients with STEMI and hypoglycemia was almost unchanged during the follow-up period (Table 1). The mean age (±SD) dropped from 67 ± 15 in 2005 to 63 ± 12 in 2017 (*p* = 0.046). Among age categories, the percentage of patients aged 55-64 significantly increased with time, whereas patients older than 84 years decreased. The prevalence of CAD decreased from 56.3% in 2005 to 53% in 2017 (*p* = 0.005), whereas dyslipidemia followed an upward trend (*p* = 0.008). However, there were no changes in the temporal trend of the rest of the cardiovascular risk factors, including diabetes. Ventricular tachycardia, ventricular fibrillation, acute renal failure, and cardiogenic shock increased with time (*p* < 0.05 for all). Acute heart failure developed in at least 20% of patients, but no temporal trend was observed. The number of patients who had primary coronary intervention (PCI) increased by almost 2-fold between 2005 and 2017 (*p* < 0.001), but the prevalence of coronary artery bypass graft (CABG) and thrombolysis were unchanged.

**TABLE 1:** Baseline characteristics, outcomes, and temporal trend of patients hospitalized for STEMI with a secondary diagnosis of hypoglycemia in the NIS database, between 2005 and 2017.

	2005	2006	2007	2008	2009	2010	2011	2012	2013	2014	2015	2016	2017	*P*-value
**Total cases (weighted)**	229	242	212	275	212	153	213	195	245	235	270	290	330	0.080
**Age**														
Mean age (SD)	67 (15)	67 (15)	67 (13)	67 (16)	69 (15)	68 (15)	63 (16)	68 (13)	67 (13)	66 (11)	64 (14)	64 (14)	63 (12)	0.046
<55	58 (25.6%)	62 (25.6%)	50 (23.6%)	66 (23.9%)	41 (19.3%)	37 (24.2%)	64 (30.2%)	40 (20.5%)	35 (14.3%)	35 (14.9%)	65 (24.1%)	60 (20.7%)	80 (24.2%)	0.244
55–64	34 (15.0%)	48 (19.8%)	64 (23.2%)	64 (23.2%)	38 (17.9%)	31 (20.3%)	63 (29.7%)	50 (25.6%)	80 (32.7%)	80 (34.0%)	80 (29.6%)	90 (31.0%)	70 (21.2%)	0.015
65–74	57 (25.1%)	33 (13.6%)	49 (17.8%)	49 (17.8%)	41 (19.3%)	31 (20.3%)	38 (17.9%)	25 (12.8%)	45 (18.4%)	65 (27.7%)	55 (20.4%)	55 (19.0%)	120 (36.4%)	0.139
75–84	38 (16.7%)	49 (20.2%)	51 (18.5%)	51 (18.5%)	53 (25.0%)	15 (9.8%)	9 (4.2%)	50 (25.6%)	45 (18.4%)	30 (12.8%)	30 (11.1%)	50 (17.2%)	45 (13.6%)	0.357
>84	40 (17.6%)	50 (20.7%)	46 (16.7%)	46 (16.7%)	39 (18.4%)	39 (25.5%)	38 (17.9%)	30 (15.4%)	40 (16.3%)	25 (10.6%)	40 (14.8%)	35 (12.1%)	15 (4.5%)	0.009
**Gender**														
Male	142 (62.0%)	169 (69.8%)	110 (51.9%)	189 (68.7%)	108 (51.2%)	92 (60.5%)	125 (58.7%)	130 (66.7%)	165 (67.3%)	165 (70.2%)	185 (68.5%)	195 (67.2%)	220 (66.7%)	0.163
Female	87 (38.0%)	73 (31.2%)	102 (48.1%)	86 (31.3%)	104 (48.8%)	61 (39.5%)	88 (41.3%)	65 (33.3%)	80 (32.7%)	70 (29.8%)	85 (31.5%)	95 (32.8%)	110 (33.3%)	0.163
**Race**														
Caucasian	141 (79.2%)	128 (78.0%)	127 (72.6%)	173 (77.9%)	116 (58.9%)	110 (78.0%)	134 (76.6%)	120 (63.2%)	145 (60.4%)	175 (83.3%)	175 (68.6%)	195 (70.9%)	240 (75.0%)	0.998
African American	13 (7.3%)	19 (11.6%)	42 (24.0%)	<11* (2.3%)	36 (18.3%)	<11* (7.1%)	18 (10.3%)	20 (10.5%)	35 (14.6%)	15 (7.1%)	55 (21.6%)	35 (12.7%)	55 (17.2%)	0.463
Hispanic	19 (10.7%)	<11* (5.5%)	<11* (0.0%)	<11* (2.3%)	14 (7.1%)	11 (7.8%)	18 (10.3%)	20 (10.5%)	30 (12.5%)	10 (4.8%)	0 (0.0%)	20 (7.3%)	15 (4.7%)	0.918
Asian	0 (0.0%)	<11* (2.4%)	0 (0.0%)	<11* (2.3%)	16 (8.1%)	<11* (3.5%)	0 (0.0%)	<11* (2.6%)	<11* (4.2%)	<11* (2.4%)	<11* (2.0%)	0 (0.0%)	<11* (1.6%)	0.931
Native American	0 (0.0%)	0 (0.0%)	0 (0.0%)	<11* (2.3%)	<11* (5.1%)	0 (0.0%)	0 (0.0%)	0 (0.0%)	0 (0.0%)	0 (0.0%)	0 (0.0%)	0 (0.0%)	0 (0.0%)	0.423
Other	<11* (2.8%)	<11* (2.4%)	<11* (3.4%)	29 (13.1%)	<11* (2.5%)	<11* (3.5%)	<11* (2.9%)	25 (13.2%)	20 (8.3%)	<11* (2.4%)	20 (7.8%)	25 (9.1%)	<11* (1.6%)	0.579
**Income**														
Low	69 (30.1%)	69 (29.6%)	77 (36.3%)	80 (30.2%)	75 (37.3%)	61 (41.2%)	57 (26.8%)	40 (21.1%)	75 (31.3%)	40 (17.8%)	85 (32.1%)	80 (28.6%)	115 (34.8%)	0.487
Low–mid	69 (30.1%)	79 (33.9%)	51 (24.1%)	76 (28.7%)	34 (16.9%)	41 (27.7%)	51 (23.9%)	70 (36.8%)	65 (27.1%)	60 (26.7%)	45 (17.0%)	75 (26.8%)	85 (25.8%)	0.404
High–mid	38 (16.6%)	72 (30.9%)	57 (26.9%)	55 (20.8%)	53 (26.4%)	21 (14.2%)	48 (22.5%)	35 (18.4%)	75 (31.3%)	55 (24.4%)	75 (28.3%)	75 (26.8%)	80 (24.2%)	0.461
High	53 (23.1%)	13 (5.6%)	27 (12.7%)	54 (20.4%)	39 (19.4%)	25 (16.9%)	57 (26.8%)	45 (23.7%)	25 (10.4%)	70 (31.1%)	60 (22.6%)	50 (17.9%)	50 (15.2%)	0.448
**Expected primary payer**														
Medicare	146 (63.8%)	137 (56.6%)	124 (58.2%)	150 (54.5%)	122 (57.8%)	75 (49.3%)	76 (35.7%)	110 (56.4%)	120 (49.0%)	120 (51.1%)	125 (46.3%)	145 (50.0%)	185 (56.1%)	0.122
Medicaid	15 (6.6%)	15 (6.2%)	0 (0.0%)	<11* (3.6%)	15 (7.1%)	<11* (3.3%)	24 (11.3%)	<11* (2.6%)	15 (6.1%)	15 (6.4%)	40 (14.8%)	40 (13.8%)	60 (18.2%)	0.009
Private insurance	63 (27.5%)	77 (31.8%)	74 (34.7%)	69 (25.1%)	58 (27.5%)	50 (32.9%)	97 (45.5%)	50 (25.6%)	65 (26.5%)	80 (34.0%)	70 (25.9%)	80 (27.6%)	75 (22.7%)	0.428
Self pay	0 (0.0%)	13 (5.4%)	15 (7.0%)	25 (9.1%)	16 (7.6%)	16 (10.5%)	16 (7.5%)	15 (7.7%)	30 (12.2%)	<11* (2.1%)	25 (9.3%)	15 (5.2%)	<11* (3.0%)	0.816
No charge	0 (0.0%)	0 (0.0%)	0 (0.0%)	0 (0.0%)	0 (0.0%)	0 (0.0%)	0 (0.0%)	0 (0.0%)	<11* (2.0%)	0 (0.0%)	0 (0.0%)	0 (0.0%)	0 (0.0%)	0.615
Other	<11* (2.2%)	0 (0.0%)	0 (0.0%)	21 (7.6%)	0 (0.0%)	<11* (3.9%)	0 (0.0%)	15 (7.7%)	<11* (4.1%)	15 (6.4%)	<11* (3.7%)	<11* (3.4%)	0 (0.0%)	0.519
**Comorbidities**														
CAD	129 (56.3%)	111 (45.9%)	116 (54.7%)	148 (53.8%)	138 (65.1%)	86 (56.2%)	115 (54.2%)	140 (71.8%)	175 (71.4%)	145 (61.7%)	155 (57.4%)	175 (60.3%)	175 (53.0%)	0.005
Diabetes	10 (4.4%)	24 (9.9%)	24 (11.3%)	10 (3.6%)	15 (7.1%)	0 (0.0%)	24 (11.3%)	15 (7.7%)	15 (6.1%)	10 (4.3%)	20 (7.4%)	20 (6.9%)	20 (6.1%)	0.788
Obesity	<11* (2.2%)	20 (8.3%)	<11* (2.4%)	16 (5.8%)	29 (13.7%)	29 (19.1%)	23 (10.8%)	20 (10.3%)	20 (8.2%)	40 (17.0%)	20 (7.4%)	15 (5.2%)	35 (10.6%)	0.34
Hypertension	129 (56.3%)	111 (45.9%)	116 (54.7%)	148 (53.8%)	138 (65.1%)	86 (56.2%)	115 (54.2%)	140 (71.8%)	175 (71.4%)	145 (61.7%)	155 (57.4%)	175 (60.3%)	175 (53.0%)	0.267
Smoking	58 (25.4%)	112 (46.3%)	68 (32.1%)	124 (45.1%)	43 (20.4%)	66 (43.1%)	81 (38.0%)	80 (41.0%)	105 (42.9%)	90 (38.3%)	115 (42.6%)	110 (37.9%)	200 (60.6%)	0.079
Dyslipidemia	77 (33.8%)	86 (35.5%)	88 (41.5%)	84 (30.5%)	81 (38.2%)	45 (29.6%)	86 (40.6%)	85 (43.6%)	115 (46.9%)	130 (55.3%)	130 (48.1%)	125 (43.1%)	150 (45.5%)	0.008
PVD	22 (9.6%)	23 (9.5%)	25 (11.8%)	44 (15.9%)	<11* (2.4%)	<11* (6.6%)	15 (7.0%)	45 (23.1%)	30 (12.2%)	30 (12.8%)	20 (7.4%)	40 (13.8%)	40 (12.1%)	0.53
Acute renal failure	27 (11.8%)	46 (19.0%)	22 (10.4%)	60 (21.8%)	30 (14.2%)	29 (19.0%)	23 (10.8%)	55 (28.2%)	25 (10.2%)	45 (19.1%)	75 (27.8%)	40 (13.8%)	50 (15.2%)	0.515
**Hospital bedsize**														
Small	<11* (20.8%)	13 (25.5%)	<11* (11.6%)	<11* (7.3%)	<11* (15.0%)	<11* (6.7%)	<11* (6.7%)	<11* (5.1%)	<11* (18.4%)	<11* (12.8%)	<11* (13.0%)	<11* (6.9%)	12 (18.2%)	0.369
Medium	<11* (16.7%)	<11* (19.6%)	11 (25.6%)	14 (25.5%)	<11* (20.0%)	<11* (23.3%)	<11* (11.1%)	<11* (15.4%)	14 (28.6%)	15 (31.9%)	14 (25.9%)	15 (25.9%)	18 (27.3%)	0.121
Large	30 (62.5%)	28 (54.9%)	27 (62.8%)	37 (67.3%)	26 (65.0%)	21 (70.0%)	37 (82.2%)	31 (79.5%)	26 (53.1%)	26 (55.3%)	33 (61.1%)	39 (67.2%)	36 (54.5%)	0.741
**Hospital location**														
Rural	<11* (10.4%)	<11* (11.8%)	<11* (11.6%)	<11* (9.1%)	<11* (12.5%)	<11* (20%)	<11* (8.9%)	<11* (10.3%)	<11* (12.2%)	<11* (4.3%)	<11* (9.3%)	<11* (5.2%)	<11* (4.5%)	0.063
Urban	43 (89.6%)	45 (88.2%)	38 (88.4%)	50 (90.9%)	35 (87.5%)	24 (80.0%)	41 (91.1%)	35 (89.7%)	43 (87.8%)	45 (95.7%)	49 (90.7%)	55 (94.8%)	63 (95.5%)	0.063
**Hospital region**														
Northeast	<11* (20.8%)	<11* (11.8%)	<11* (9.3%)	<11* (14.5%)	<11* (4.7%)	<11* (6.7%)	<11* (13.3%)	<11* (12.8%)	<11* (6.1%)	<11* (17.0%)	<11* (13.0%)	<11* (8.6%)	<11* (15.2%)	0.782
Midwest	<11* (16.7%)	<11* (15.7%)	<11* (18.6%)	<11* (18.2%)	<11* (18.6%)	<11* (23.3%)	<11* (17.8%)	11 (28.2%)	15 (30.6%)	11 (23.4%)	17 (31.5%)	16 (27.6%)	18 (27.3%)	<0.001
South	21 (43.8%)	21 (41.2%)	26 (60.5%)	25 (45.5%)	16 (37.2%)	15 (50.0%)	18 (40.0%)	14 (35.9%)	21 (42.9%)	18 (38.3%)	21 (38.9%)	21 (36.2%)	28 (42.4%)	0.114
West	<11* (18.8%)	16 (31.4%)	<11* (11.6%)	12 (21.8%)	17 (39.5%)	<11* (20.0%)	13 (28.9%)	<11* (23.1%)	<11* (20.4%)	<11* (21.3%)	<11* (16.7%)	16 (27.6%)	10 (15.2%)	0.594
LoS	3 (4)	4 (7)	3 (4)	3 (5)	4 (7)	5.5 (7)	5 (6.5)	6 (10)	4 (7)	4 (6)	5 (8)	5 (7)	5 (8)	0.004
**Outcomes**														
Mortality	61 (26.8%)	54 (22.4%)	64 (30.2%)	106 (38.5%)	79 (37.3%)	41 (27.0%)	33 (15.5%)	75 (38.5%)	80 (32.7%)	70 (29.8%)	80 (29.6%)	100 (34.5%)	135 (40.9%)	0.234
Ventricular tachycardia	32 (14.0%)	<11* (3.7%)	22 (10.4%)	30 (10.9%)	<11* (4.3%)	20 (13.1%)	28 (13.1%)	30 (15.4%)	35 (14.3%)	35 (14.9%)	40 (14.8%)	60 (20.7%)	65 (19.7%)	0.004
Ventricular fibrillation	15 (6.6%)	5 (2.1%)	15 (7.1%)	20 (7.3%)	29 (13.7%)	11 (7.2%)	23 (10.8%)	35 (17.9%)	65 (26.5%)	40 (17.0%)	60 (22.2%)	65 (22.4%)	80 (24.2%)	<0.001
Atrial fibrillation	28 (12.3%)	33 (13.6%)	32 (15.1%)	57 (20.7%)	42 (19.9%)	30 (19.6%)	58 (27.2%)	55 (28.2%)	50 (20.4%)	20 (8.5%)	55 (20.4%)	90 (31.0%)	90 (27.3%)	0.541
Acute heart failure	47 (20.6%)	80 (33.1%)	51 (24.1%)	73 (26.5%)	106 (50.2%)	59 (38.8%)	69 (32.4%)	95 (48.7%)	65 (26.5%)	45 (19.1%)	105 (38.9%)	105 (36.2%)	140 (42.4%)	0.303
Ischemic stroke	5 (2.2%)	<11* (3.7%)	12 (5.7%)	0 (0.0%)	0 (0%)	<11* (3.3%)	<11* (2.3%)	<11* (2.6%)	0 (0.0%)	<11* (2.1%)	0 (0.0%)	15 (5.2%)	15 (4.5%)	0.888
Acute renal failure	23 (10.0%)	24 (9.9%)	44 (20.8%)	78 (28.4%)	74 (35.1%)	36 (23.7%)	56 (26.3%)	70 (35.9%)	70 (28.6%)	90 (38.3%)	115 (42.6%)	105 (36.2%)	175 (53.0%)	<0.001
Cardiogenic shock	<11* (3.9%)	15 (6.2%)	11 (5.2%)	34 (12.4%)	78 (37%)	36 (23.7%)	42 (19.8%)	80 (41.0%)	65 (26.5%)	80 (34.0%)	100 (37.0%)	110 (37.9%)	110 (33.3%)	0.001
**Interventions**														
PCI	82 (35.8%)	81 (33.5%)	65 (30.7%)	133 (48.2%)	82 (38.9%)	61 (40.1%)	117 (55.2%)	80 (41.0%)	140 (57.1%)	140 (59.6%)	165 (61.1%)	200 (70.0%)	225 (68.2%)	<0.001
Thrombolysis	<11* (2.2%)	<11* (2.1%)	<11* (1.4%)	<11* (3.6%)	0 (0%)	0 (0%)	0 (0%)	0 (0%)	<11* (4.1%)	<11* (2.1%)	<11* (1.9%)	0 (0%)	0 (0%)	0.385
CABG	24 (10.5%)	68 (28.2%)	20 (9.4%)	35 (12.7%)	14 (6.6%)	36 (23.7%)	49 (23.0%)	50 (25.6%)	50 (20.4%)	40 (17.0%)	25 (9.3%)	30 (10.3%)	25 (7.6%)	0.549

CABG, coronary artery bypass graft; CAD, coronary artery disease; LoS, length of stay; PCI, percutaneous coronary intervention; PVD, peripheral vascular disease.

*Per the HCUP’s requirements, cells less or equal to 10 are noted as < 11.

A drop in age was also observed in STEMI patients without hypoglycemia (66 ± 14 in 2005 to 66 ± 13 in 2017, *p* < 0.001) (Supplementary Table 1). All cardiovascular risk factors, including diabetes, followed a statistically significant upward trend. The prevalence of ventricular tachycardia, ventricular fibrillation, acute renal failure, and cardiogenic shock increased during observation, concordant with findings reported in STEMI patients with hypoglycemia. However, mortality decreased from 9.6 to 7.9% (*p* < 0.001).

**FIGURE 2:**
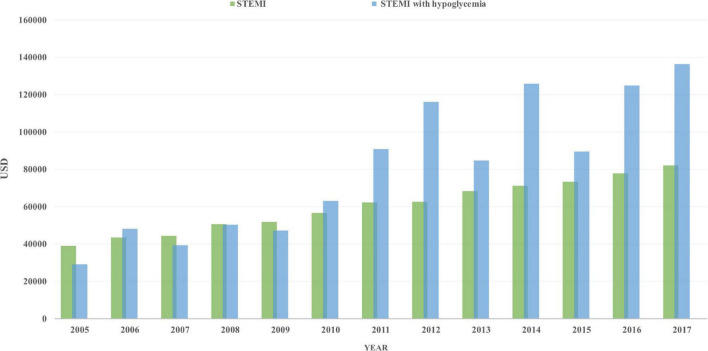
Total charges/stay (after adjustment for inflation) in STEMI patients with hypoglycemia (blue) and those without hypoglycemia (green), from 2005 to 2017.

Total charges increased in both groups, from 29,140 to 136,453 USD/stay in STEMI patients with hypoglycemia and from 39,032 to 82,134 USD/stay in those without hypoglycemia (Figure 2). Interestingly, the length of stay (LoS) increased from 3 (4) to 5 (8) in patients with hypoglycemia (*p* = 0.004) but was unchanged in those without hypoglycemia.

### Comparison of baseline characteristics and outcomes of both groups

#### Trend

We combined all STEMI patients with hypoglycemia from 2005 to 2017, then compared them to those without hypoglycemia for the same period. As seen in Table 2, patients with hypoglycemia were, on average, two years older (66 vs. 64 years old in STEMI without hypoglycemia), with a higher proportion of patients >84 years old (*p* < 0.001 for both). The male gender was dominant in both. Patients with STEMI and hypoglycemia were more likely Blacks and less likely to be Whites and private insurance (*p* < 0.001 for all). Surprisingly, only 6.7% of patients who developed hypoglycemia had diabetes compared to 28.5% of those who didn’t develop hypoglycemia (*p* < 0.001). Obesity, hypertension, and dyslipidemia were less prevalent in the hypoglycemia group, while renal failure and PVD were more prevalent.

**TABLE 2:** Comparison of baseline characteristics of STEMI patients according to the presence of hypoglycemia.

	STEMI	STEMI + Hypoglycemia	*P*-value
**Age**			
Mean (SD)	64 (14)	66 (14)	0.001
<55	142,791 (26.7%)	140 (22.3%)	0.627
55–64	140,083 (26.2%)	156 (24.8%)	0.274
65–74	112,982 (21.2%)	131 (20.9%)	0.168
75–84	86,076 (16.1%)	106 (16.9%)	0.077
>84	52,007 (9.7%)	95 (15.1%)	<0.001
**Gender**			
Male	354,577 (66.4%)	403 (64.2%)	0.345
Female	179,362 (33.6%)	225 (35.8%)	0.236
**Race**			
Caucasian	351,689 (78.3%)	401 (72.1%)	0.005
African American	35,913 (8.0%)	73 (13.1%)	<0.001
Hispanic	33,164 (7.4%)	35 (6.3%)	0.661
Asian	10,248 (2.3%)	12 (2.2%)	0.928
Native American	2,569 (0.6%)	3 (0.5%)	0.967
Other	15,730 (3.5%)	32 (5.8%)	0.002
**Income**			
Low	147,530 (28.3%)	187 (30.5%)	0.198
Low-Mid	143,156 (27.5%)	162 (26.4%)	0.291
High-Mid	126,179 (24.2%)	150 (24.4%)	0.559
High	104,255 (20.0%)	115 (18.7%)	0.241
**Primary expected payer**			
Medicare	247,793 (46.5%)	332 (52.9%)	0.032
Medicaid	38,443 (7.2%)	52 (8.3%)	0.949
Private Insurance	183,252 (34.4%)	185 (29.5%)	0.002
Self Pay	41,265 (7.7%)	40 (6.4%)	0.053
No Charge	3,840 (0.7%)	1 (0.2%)	0.102
Other	18,204 (3.4%)	18 (2.9%)	0.21
**Comorbidities**			
CAD	356,296 (66.7%)	330 (52.5%)	<0.001
Diabetes	151,960 (28.5%)	42 (6.7%)	<0.001
Obesity	62,887 (11.8%)	56 (8.9%)	0.027
Hypertension	307,590 (57.6%)	323 (51.4%)	0.002
Smoking	195,890 (36.7%)	207 (33.0%)	0.053
Dyslipidemia	294,719 (55.2%)	256 (40.8%)	<0.001
PVD	41,188 (7.7%)	71 (11.3%)	0.001
Renal Failure	54,463 (10.2%)	107 (17.0%)	<0.001
**Hospital bedsize**			
Small	60,272 (11.3%)	83 (13.3%)	0.567
Medium	130,090 (24.5%)	145 (23.2%)	0.125
Large	341,439 (64.2%)	397 (63.5%)	0.161
**Hospital location**			
Rural	53,853 (10.1%)	59 (9.4%)	0.76
Urban	477,948 (89.9%)	566 (90.6%)	0.57
**Hospital region**			
Northeast	90,738 (17.0%)	76 (12.1%)	0.037
Midwest	126,916 (23.8%)	145 (23.1%)	0.028
South	217,051 (40.7%)	265 (42.2%)	0.004
West	99,234 (18.6%)	142 (22.6%)	< 0.001

### Comparison of both groups

**FIGURE 3:**
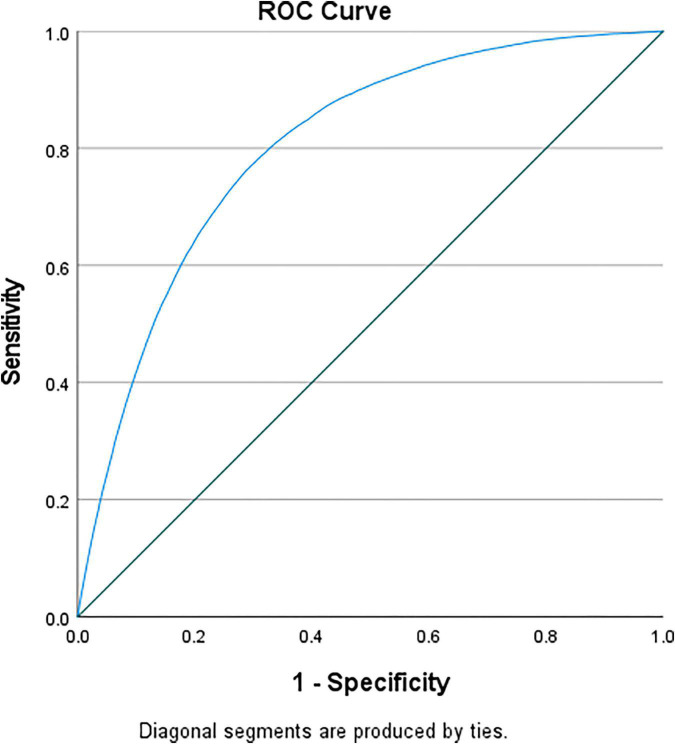
ROC curve testing the prediction of mortality of the multivariable regression model in patients with STEMI and diabetes.

**FIGURE 4:**
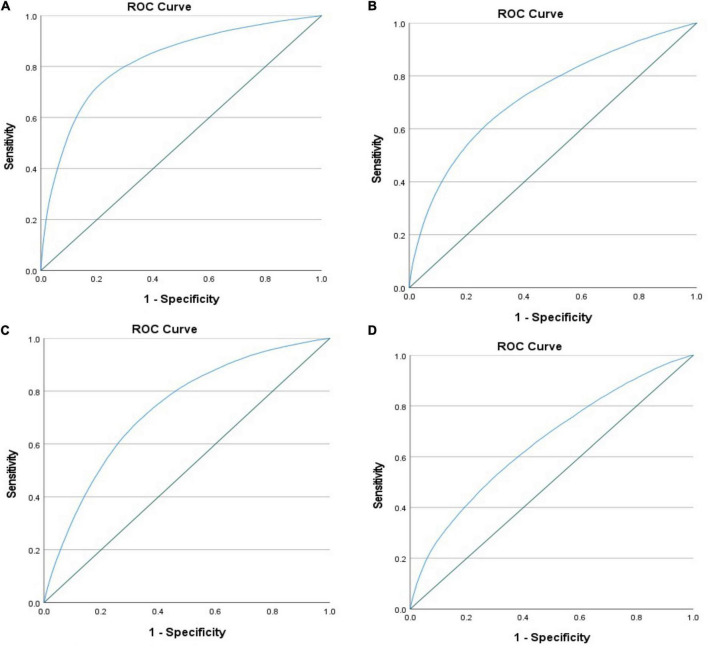
ROC curve testing the prediction of the multivariable regression model in patients with STEMI and diabetes regarding **(A)** acute renal failure, **(B)** cardiogenic shock, mortality, **(C)** atrial fibrillation, and **(D)** ventricular fibrillation.

Cardiovascular events were more likely to occur in the presence of hypoglycemia (Table 3). Mortality risk increased by almost 4-fold and remained high after multivariable adjustment (adjusted OR = 2.625 [2.095–3.289]). There was a higher incidence of cardiogenic shock (adjusted OR = 1.718 [1.387–2.127]), atrial fibrillation (adjusted OR = 1.284 [1.025–1.607]), ventricular fibrillation (adjusted OR = 1.799 [1.406–2.301]), and acute renal failure (adjusted OR = 2.355 [1.902–2.917]). The ROC curve showed that all models were adequate and fit. The multivariable model had an excellent prediction of mortality (AUC = 0.84, *p* < 0.001) (Figure 3) and acute renal failure (AUC = 0.82, *p* < 0.001) (Figure 4A), a good prediction for cardiogenic shock (AUC = 0.72, *p* < 0.001) (Figure 4B), atrial fibrillation (AUC = 0.73, *p* < 0.001) (Figure 4C), but a moderate prediction for ventricular fibrillation (AUC = 0.65, *p* < 0.001) (Figure 4D).

**TABLE 3:** Cardiovascular outcomes and interventions STEMI patients according to the presence of hypoglycemia.

Outcome/intervention	Number of events (OR, 95% CI)		Adjusted OR (95% CI)	*P*-value
	STEMI	STEMI + Hypoglycemia		
Mortality	226211 (OR = 1)	989 (4.490; 4.174–5.848)	2.625 (2.095–3.289)	<0.001
Ventricular tachycardia	242962 (OR = 1)	415 (1.550; 1.233–1.948)	1.245 (0.971–1.595)	0.084
Ventricular fibrillation	161600 (OR = 1)	463 (2.677; 2.147–3.337)	1.799 (1.406–2.301)	0.001
Atrial fibrillation	342599 (OR = 1)	640 (1.742; 1.435–2.114)	1.284 (1.025–1.607)	0.03
Acute heart failure	605966 (OR = 1)	1040 (1.705; 1.445–2.013)	1.150 (0.942–1.404)	0.171
Stroke	34311 (OR = 1)	76 (1.865; 1.117–3.114)	1.132 (0.657–1.951)	0.656
Acute renal failure	293277 (OR = 1)	960 (3.547; 3.016–4.235)	2.355 (1.902–2.917)	<0.001
Cardiogenic shock	267842 (OR = 1)	770 (2.914; 2.430–3.494)	1.718 (1.387–2.127)	<0.001
PCI	1791655 (OR = 1)	1571 (0.488; 0.417–0.571)	0.596 (0.491–0.722)	<0.001
Thrombolysis	57815 (OR = 1)	43 (0.647; 0.335–1.249)	0.689 (0.342–1.387)	0.297
CABG	190460 (OR = 1)	466 (2.259; 1.814–2.814)	1.792 (1.391–2.308)	<0.001

A higher incidence of ventricular tachycardia, acute heart failure, and stroke was observed in STEMI patients with hypoglycemia, but events were not statistically significant after adjustment. Patients with STEMI and diabetes were less likely to have PCI (OR = 0.596 [0.491–0.722]) but more likely to have CABG (OR = 1.792 [1.391–2.308]). Total charges/stay were higher in patients with hypoglycemia than those without (80,466 vs. 60,319 USD; respectively; *p* < 0.001). Unsurprisingly, patients who developed hypoglycemia had a slightly longer mean LoS (4[3] vs. 3[3] days, hypoglycemia versus non-hypoglycemia, *p* = 0.01).

### Predictors of mortality in STEMI patients with hypoglycemia

We further looked for predictors of mortality in STEMI patients with hypoglycemia. As expected, old age is associated with higher mortality risk. For instance, patients over 84 years have an almost 4.5-fold higher risk of dying than those 55 years of age or younger (Table 4). Females are more predisposed than males (OR = 1.711 [1.211–2.418]), and Blacks have a doubled risk as compared to Whites (OR = 1.965 [1.179–3.274]). Valvular heart disease, renal failure, and peripheral vascular disease – but not coronary artery disease- increased the risk significantly. Interestingly, cardiometabolic risk factors such as obesity, hypertension, dyslipidemia, and smoking were associated with lower mortality risk. Surprisingly, diabetes did not predict mortality (OR = 0.757 [0.372–1.539]). To validate our findings, we tested the interaction between diabetes and hypoglycemia and did not observe any (p interaction = 0.318).

**TABLE 4:** Predictors of mortality in STEMI patients with hypoglycemia.

	OR (95% CI)	*P*-value
**Age**		
<55	Ref	Ref
55–64	1.288 (0.741–2.238)	0.369
65–74	2.238 (1.295–3.867)	0.004
75–84	1.972 (1.105–3.519)	0.022
>84	4.444 (2.494–7.919)	<0.001
**Gender**		
Male	Ref	Ref
Female	1.711 (1.211–2.418)	0.002
**Race**		
Caucasian	Ref	Ref
African American	1.965 (1.179–3.274)	0.01
Hispanic	0.629 (0.267–1.482)	0.289
Asian	0.839 (0.223–3.156)	0.795
Native American	1.259 (0.113–14.019	0.852
Other	1.958 (0.942–4.069)	0.072
**Income**		
Low	1	Ref
Low-Mid	0.609 (0.386 -0.960)	0.033
High-Mid	0.700 (0.443–1.106)	0.127
High	0.658 (0.399–1.085)	0.101
**Primary expected payer**		
Medicare	Ref	Ref
Medicaid	0.929 (0.507–1.704)	0.813
Private Insurance	0.377 (0.245–0.579)	<0.001
Self Pay	0.612 (0.296–1.269)	0.187
No Charge	2.608E + 9 (0–0.)	1
Other	0.621 (0.216–1.783)	0.376
**Comorbidities**		
CAD	0.329 (0.229–0.473)	<0.001
Diabetes	0.757 (0.372–1.539)	0.442
Obesity	0.632 (0.332–1.205)	0.164
Hypertension	0.768 (0.547–1.079)	0.128
Smoking	0.656 (0.461–0.932)	0.019
Dyslipidemia	0.345 (0.237–0.501)	<0.001
PVD	1.124 (0.666–1.897)	0.662
Renal Failure	1.586 (1.032–2.438)	0.035
**Hospital bed size**		
Small	Ref	Ref
Medium	1.334 (7.44–2.392)	0.334
Large	1.091 (0.648–1.835)	0.744
**Hospital location**		
Rural	Ref	Ref
Urban	0.695 (0.400–1.208)	0.197
**Hospital region**		
Northeast	Ref	Ref
Midwest	1.009 (0.559–1.822)	0.975
South	0.997 (0.579–1.716)	0.992
West	0.745 (0.407–1.366)	0.342

## Discussion

Hypoglycemia is consistently associated with mortality in critically ill patients admitted to the intensive care unit. In a retrospective analysis of US and Dutch cohorts, Krinsley et al. reported an excess of hospital mortality in ICU patients who developed hypoglycemia (11). Those findings were confirmed in an Australian study of almost 5,000 ICU patients. Interestingly, the mortality risk was significantly higher even in patients with mild to moderate hypoglycemia (12). In the NICE-SUGAR study, tight glucose control in intensive care patients led to more hypoglycemic events and higher mortality (13). A higher incidence of hypoglycemia was also reported by the investigators of the CHiP study in pediatric critically ill patients (14).

Data assessing the cardiovascular outcomes of hypoglycemia in STEMI is missing. To our knowledge, we are the first to explore trends across risk factors and outline excess mortality and morbidity in STEMI patients irrespective of diabetes. However, it has already been shown in smaller-scale experimental models showed that hypoglycemia is associated with ischemic changes in patients with diabetes. Using a 3-day continuous glucose monitoring in 19 patients with diabetes and CAD, Desouza et al. reported chest pain and non-specific ECG changes on Holter monitoring during hypoglycemia (15). Similar studies revealed the presence of ectopic atrial beats and QTc prolongation; findings were confirmed in a meta-analysis that included 15 of those studies (16). Changes in the ST segment have also been reported in case reports from Israel (17) and the United Kingdom (18).

Our data aligns with several epidemiological cohorts and *ad hoc* analyses of trials that showed an increased mortality risk with hypoglycemia in the non-ICU diabetes population. Earlier in the ’70s, a British cohort reported an excess mortality risk of 4% with hypoglycemia in diabetes patients under 50 years of age (19). In a recent Norwegian study, the mortality risk was 8% in type 1 diabetes patients younger than 56 years old of age (20). In the ORIGIN trial that included over 12,000 patients with type 2 diabetes, mortality risk was increased by almost 2-fold in the presence of severe hypoglycemia. Similar findings have been reported with type 2 diabetes patients in the DEVOTE 3 and EXAMINE trials (21, 22). In a recent *post hoc* analysis of the ACCORD study, severe hypoglycemia increased the risk of heart failure by almost 50% (23).

Strict glycemic control in patients with diabetes, particularly in long-standing diabetes with complications, is not recommended by the American Diabetes Association and similar cardiac societies for almost a decade now (24, 25). Three large trials—ACCORD (26), VADT (27), and ADVANCE (28)—failed to show any survival benefit with an intensive glycemic control approach in high-risk patients. Further, the ACCORD study revealed a deleterious effect of aggressive anti-hyperglycemic therapy as mortality increased. Furthermore, the three studies showed an association between hypoglycemic events and mortality, although causality was not established. In a meta-analysis that regrouped those trials and the older UKPDS study, Kelly et al. showed that intensive treatment did not reduce the risk of all-cause or cardiovascular mortality but increased episodes of severe hypoglycemia (29). However, newer anti-diabetes medications are safer (30). DDP-IV inhibitors improve HBA_1*C*_ without inducing hypoglycemia; nevertheless, they do not reduce cardiovascular events in the long term (31). GLP-1 agonists do not cause hypoglycemia when administered to non-diabetes individuals or those with diabetes not otherwise treated with other classes (32, 33). However, hypoglycemic episodes have been reported when GLP-1 agonists were combined with insulin or sulfonylureas (34), which is usually handled by decreasing the dose of the latter two classes.

Similarly, SGLT-2 inhibitors decrease glycemia by blocking the reabsorption of filtered glucose in the presence of hyperglycemia. However, the excretion of glucose almost ceases in the presence of normoglycemia (35). Thus, they do not usually induce hypoglycemia in the absence of therapies that otherwise cause hypoglycemia.

It is not entirely understood why hypoglycemia increases cardiovascular risk. Further, a clear causality role between hypoglycemia and cardiovascular events could not be established. In the ADVANCE trial, repeated episodes of severe hypoglycemia did not correlate with the cardiovascular outcome; hence, hypoglycemia could have been only a marker of vulnerability (36). Nevertheless, several experimental studies suggest a direct novice effect of hypoglycemia on endothelial function, cardiac repolarization, and blood coagulability. Hypoglycemia activates the sympathoadrenal system, which results in a release of catecholamines. In experimental models, catecholamines induce blood coagulation abnormalities, including platelets and factor VII activation, resulting in a pro-inflammatory status mediated by CRP, VEGF, and interleukin 6 (37). Rana et al. reported a decreased myocardial blood flow reserve in healthy volunteers and type 1 diabetes subjects following a hypoglycemic clamp (38). Human umbilical vein endothelial cells exposed to hypoglycemia exhibit decreased nitric oxide and increased superoxide, both pathways leading to endothelial dysfunction (39).

Interestingly, hypoglycemia or related mortality did not decrease during the observation study. However, hospitalizations and mortality decreased in STEMI patients without hypoglycemia, which is concordant with the current bibliography pertinent to cardiovascular disease trends and patterns. An average 3% yearly reduction in MI-related mortality was reported in a 10-year analysis of the Medicare (40). We have recently shown that diabetes-related mortality is in a descending slot in patients with heart failure (41), stroke (42), and aortic valve replacement (43) despite the increase in the prevalence of diabetes and associated risk factors. Surprisingly, some of the classically harmful cardiometabolic parameters, such as hypertension, dyslipidemia, and smoking, were associated with decreased mortality in our study, findings reported in previous cardiovascular studies from the NIS pertinent to myocardial infarction, heart failure, stroke, and aortic valve replacement (41–45).

Contrary to our expectations, smoking was associated with lower mortality risk. Although correlation does not equate to causation, one plausible mechanism behind this paradoxical finding is that smokers usually receive more cardioprotective drugs known to decrease mortality, such as anti-platelets. In a sub-analysis of the CHARISMA trial, clopidogrel reduced mortality and major cardiovascular events in current smokers but not in former or never smokers (46). It might also be possible that smokers are relatively younger and have fewer cardiovascular comorbidities on admission, which has been recently reported in ST-elevation myocardial infarction patients who had lower mortality risk when undergoing PCI (47).

We acknowledge the presence of limitations in this study. Our cohort is an administrative database that was not designed to assess cardiovascular events or show causality. The time-to-first event is missing in the NIS database, making creating a Cox proportional hazards regression model, rather than a multivariable logistic regression model, impossible. It might also be possible that the multivariable logistic regression model subjects the same data set to multiple testing, increasing the risk of false positives. Other important parameters of hypoglycemia are missing, such as baseline medications, glycemic control, and diabetes duration, knowing that those factors are strong predictors of hypoglycemia-related mortality; hence, we could not account for those factors in our regression model. Further, it is unknown whether hypoglycemic events were symptomatic or silent, nor their severity. Finally, all diagnoses and outcomes were based on ICD-9 and ICD-10, so we cannot exclude erroneous coding or misclassification, including in 2015 when the transition between the 9^th^ and 10^th^ versions occurred.

## Conclusion

In this large observational cohort, the prevalence of hypoglycemia in patients hospitalized for ST-elevation myocardial infarction is rare. Nevertheless, it was associated with increased mortality, atrial fibrillation, ventricular fibrillation, and acute renal failure, irrespective of diabetes at baseline. Although our results need to be duplicated in different cohorts, particularly cardiac cohorts of myocardial infarction, the findings illustrated in this work stress the importance of avoiding hypoglycemia in patients presenting with an acute coronary syndrome, particularly in vulnerable patients. With mounting data supporting the deleterious effect of hyperglycemia and hypoglycemia in critically ill cardiac patients, reaching normoglycemia is an important goal that cardiologists and endocrinologists should aim for.

## Data availability statement

All datasets generated for this study are included in the article/Supplementary material.

## Ethics statement

The study received administrative IRB approval from Weill Cornell Medicine-Qatar as it contains only de-identified data (record number 18-00017). Written informed consent for participation was not required for this study in accordance with the national legislation and the institutional requirements.

## Author contributions

CAK made conception and design and was the guarantor of this work and, as such, has full access to all the data in the study and takes responsibility for the integrity of the data and the accuracy of the data analysis. BH made acquisition of data and prepared figures. ZM and SD performed the statistical analyses. BH, JA, HJ, and CAK made analysis and interpretation of the data. BH and CAK wrote the manuscript. All authors read and approved the final manuscript.
