# Supplementary material for: Hypoglycemia is associated with a higher risk of mortality and arrhythmias in ST-elevation myocardial infarction, irrespective of diabetes
Source: Front Cardiovasc Med. 2022 Oct 10;9:940035. doi: 10.3389/fcvm.2022.940035 (PMC9588908; doi:10.3389/fcvm.2022.940035)
Supplement: Supplementary file 1 [file Data_Sheet_1.docx]

**Supplemental Table 1**: ICD 9 and ICD 10 codes used in the study

| Outcomes | ICD9 Codes | ICD10 Codes |
| --- | --- | --- |
| STEMI code | All 410 except 410.7 and its subgroups (70,71 and 72) | I21.0, I21.2, I21.3. |
| hypoglycemia | ICD9: 251.0, 251.1, 251.2, 962.3 | ICD10: E15, E16.0, E16.1, E16.2, T38.3X1A, T38.3X2A, T38.3X3A, T38.3X4A |
| Ventricular tachycardia | 427.1 | I47.2 |
| Ventricular fibrillation | 427.41 | I49.01 |
| Atrial fibrillation | 427.31 | I48.91 |
| Acute Heart failure | 402.01, 402.11, 402.91, 404.01, 404.03, 404.11, 404.13, 404.91, 404.93, **and all 428** | I11.0, I13.0, I13.2, I50.814, I50.9, I150.1, I150.20, (I150.21 to 23), (I150.40 to 43), (I150.810 to 813), (I150.82 to 84), I1590.89, I50.9 + I50.30+I50.31+I50.32+I50.33 |
| stroke | 434.01, 434.11, 434.91 , 433.01, 433.11, 433.21, 433.31, 433.81, 433.91, 433.01, 433.11, 433.21, 433.31, 433.81, 433.91, 436 | I63.30, I63.40, I63.50, I63.22, I63.139, I63.239, I63.019, I63.119, I63.219, I63.59, I63.20, I67.89 |
| Acute renal failure | 584.5 till 584.9 | N17.0, N17.1, N17.2, N17.8, N17.9 |
| Cardiogenic shock | 785.51 | R57.0 |
| procedures outcomes |  |  |
| PCI | 00.66, 36.06, 36.07, 17.55 | 02C03ZZ, 02703ZZ,02704ZZ, 02713ZZ, 02714ZZ, 02723ZZ, 02714ZZ, 02724ZZ, 02733ZZ, 02734ZZ |
| Thrombolysis | 99.10 and V45.88 | 3E03317, 3E04317, 3E05317, 3E06317, 3E08317,0 Z92.82 |
| CABG | 36.10 to 36.19 | Aorto-coronary bypass for revascularization, not otherwise specified: 0210093, 02100A3, 02100J3, 02100K3, 02100Z3, 0210493, 02104A3, 02104J3, 02104K3, 02104Z3 |
|  |  | Aorto-coronary bypass of one coronary artery: 021008W, 021009W, 02100AW, 02100JW, 02100KW, 021048W, 021049W, 02104AW, 02104JW, 02104KW. |
|  |  | Aorto-coronary bypass of two coronary arteries: 021108W, 021109W, 02110AW, 02110JW, 02110KW, 021148W, 021149W, 02114AW, 02114JW, 02114KW. |
|  |  | Aorto-coronary bypass of three coronary arteries: 021208W, 021209W, 02120AW, 02120JW, 02120KW, 021248W, 021249W, 02124AW, 02124JW, 02124KW. |
|  |  | Aorto-coronary bypass of 4+ coronary artieris: 021308W, 021309W, 02130AW, 02130JW, 02130KW, 021348W, 021349W, 02134AW, 02134JW, 02134KW. |
|  |  | Single Internal Mammary coronary artery bypass: 0210088, 0210089, 021008C, 0210098, 0210099, 021009C, 02100A8, 02100A9, 02100AC, 02100J8, 02100J9, 02100JC, 02100K8, 02100K9, 02100KC, 02100Z8, 02100Z9, 02100ZC, 0210488, 0210489, 021048C, 0210498, 0210499, 021049C, 02104A8, 02104A9, 02104AC, 02104J8, 02104J9, 02104JC, 02104K8, 02104K9, 02104KC, 02104Z8, 02104Z9, 02104ZC |
|  |  | Double Internal Mammary coronary artery bypass: 0211088, 0211089, 021108C, 0211098, 0211099, 021109C, 02110A8, 02110A9, 02110AC, 02110J8, 02110J9, 02110JC, 02110K8, 02110K9, 02110KC, 02110Z8, 02110Z9, 02110ZC, 0211488, 0211489, 021148C, 0211498, 0211499, 021149C, 02114A8, 02114A9, 02114AC, 02114J8, 02114J9, 02114JC, 02114K8, 02114K9, 02114KC, 02114Z8, 02114Z9, 02114ZC, 021208C, 021209C, 02120AC, 02120JC, 02120KC, 02120ZC, 021248C, 021249C, 02124AC, 02124JC, 02124KC, 02124ZC, 021308C, 021309C, 02130AC, 02130JC, 02130KC, 02130ZC, 021348C, 021349C, 02134AC, 02134JC, 02134KC, 02134ZC, |
|  |  | Abdominal coronary artery bypass: 021008F, 021009F, 02100AF, 02100JF, 02100KF, 02100ZF, 021048F, 021049F, 02104AF, 02104JF, 02104KF, 02104ZF |
|  |  | Other bypass anastomosis: 0210083, 0210093, 02100A3, 02100J3, 02100K3, 02100Z3, 0210483, 0210493, 02104A3, 02104J3, 02104K3, 02104Z3 |

**Supplemental Table 2**: Baseline characteristics, outcomes and temporal trend of patients hospitalized for STEMI in the NIS database, between 2005 and 2017.

|  | **2005** | **2006** | **2007** | **2008** | **2009** | **2010** | **2011** | **2012** | **2013** | **2014** | **2015** | **2016** | **2017** | **P value** |
| --- | --- | --- | --- | --- | --- | --- | --- | --- | --- | --- | --- | --- | --- | --- |
| **Total cases (Unweighted)** | 54417 | 55229 | 46176 | 46209 | 41888 | 39326 | 39667 | 36192 | 35203 | 34505 | 35022 | 35486 | 35247 | <0.001 |
| **Total cases (Weighted)** | 265962 | 268981 | 228138 | 226773 | 210932 | 196381 | 189495 | 180960 | 176015 | 172525 | 175110 | 177430 | 176235 | <0.001 |
| **Age** |  |  |  |  |  |  |  |  |  |  |  |  |  |  |
| Mean Age (SD) | 66  (14) | 65  (14) | 65  (14) | 65  (14) | 64  (14) | 64  (14) | 64  (14) | 64  (13) | 64  (13) | 63  (13) | 63  (13) | 63  (13) | 63  (13) | <0.001 |
| <55 | 66432 (25%) | 72491 (27%) | 62387 (27.3%) | 61071 (26.9%) | 59231 (27.9%) | 54855 (27.9%) | 51916 (27.4%) | 49675 (27.5%) | 46810 (26.6%) | 46230 (26.8%) | 45430 (25.9%) | 45360 (25.6%) | 44665 (25.3%) | 0.299 |
| 55-64 | 60479 (22.7%) | 64016 (23.8%) | 55015 (24.1%) | 56493 (24.9%) | 25166 (26.6%) | 52166 (26.6%) | 51442 (27.1%) | 49420 (27.3%) | 49435 (28.1%) | 48500 (28.1%) | 50355 (28.8%) | 51505 (29.0%) | 51185 (29.0%) | <0.001 |
| 65 - 74 | 53839 (20.2%) | 52958 (19.7%) | 43917 (19.3%) | 42439 (20.1%) | 39220 (20.0%) | 39220 (20.0%) | 38790 (20.5%) | 38960 (21.5%) | 38880 (22.1%) | 39165 (22.7%) | 40445 (23.1%) | 41810 (23.6%) | 43305 (24.6%) | <0.001 |
| 75-84 | 53356 (20.1%) | 50005 (18.6%) | 40941 (17.9%) | 34143 (16.2%) | 30679 (15.6%) | 30679 (15.6%) | 28506 (15.0%) | 26735 (14.8%) | 25310 (14.4%) | 24140 (14.0%) | 24925 (14.2%) | 25045 (14.1%) | 24455 (13.9%) | <0.001 |
| >84 | 31856 (12.0%) | 29511 (11.0%) | 25879 (11.3%) | 21526 (10.2%) | 19462 (9.9%) | 19462 (9.9%) | 18841 (9.9%) | 16170 (8.9%) | 15580 (8.9%) | 14490 (8.4%) | 13955 (8.0%) | 13710 (7.7%) | 12625 (7.2%) | <0.001 |
| **Gender** |  |  |  |  |  |  |  |  |  |  |  |  |  |  |
| Male | 167147 (62.8%) | 173705 (64.6%) | 147254 (64.5%) | 147378 (65.0%) | 140161 (66.4%) | 130940 (66.7%) | 126737 (66.9%) | 121950 (67.4%) | 119270 (67.8%) | 118280 (68.6%) | 119490 (68.2%) | 121870 (68.7%) | 121850 (69.1%) | <0.001 |
| Female | 98815 (37.2%) | 95276 (35.4%) | 80884 (35.5%) | 739395 (35.0%) | 70771 (33.6%) | 65441 (33.3%) | 62758 (33.1%) | 59010 (32.6%) | 56745 (32.2%) | 54245 (31.4%) | 55620 (31.8%) | 55560 (31.3%) | 54385 (30.9%) | <0.001 |
| **Race** |  |  |  |  |  |  |  |  |  |  |  |  |  |  |
| Caucasian | 152334 (82.0%) | 154957 (81.1%) | 127203 (78.4%) | 143682 (79.7%) | 136727 (78.0%) | 131423 (77.8%) | 129724 (77.3%) | 131335 (77.8%) | 127825 (78.0%) | 125850 (77.8%) | 126540 (77.0%) | 128770 (76.7%) | 126820 (75.4%) | <0.001 |
| African American | 11285 (6.1%) | 13099 (6.9%) | 13480 (8.3%) | 13413 (7.4%) | 13686 (7.8%) | 14300 (8.5%) | 14193 (8.5%) | 14110 (8.4%) | 13290 (8.1%) | 13150 (8.1%) | 14260 (8.7%) | 14820 (8.8%) | 14850 (8.8%) | 0.001 |
| Hispanic | 13074 (7.0%) | 13170 (6.9%) | 11869 (7.3%) | 10839 (6.0%) | 11929 (6.8%) | 12537 (7.4%) | 12444 (7.4%) | 12500 (7.4%) | 12360 (7.5%) | 11850 (7.3%) | 12795 (7.8%) | 13230 (7.9%) | 14690 (8.7%) | 0.003 |
| Asian | 2915 (1.6%) | 3533 (1.9%) | 3051 (1.9%) | 3814 (2.1%) | 3861  (2.2%) | 3895 (2.3%) | 3749 (2.2%) | 3565 (2.1%) | 3985 (2.4%) | 4120 (2.5%) | 4410 (2.7%) | 4575 (2.7%) | 4885 (2.9%) | <0.001 |
| Native American | 640 (0.3%) | 1012 (0.5%) | 954  (0.6%) | 1722 (1.0%) | 1148  (0.7%) | 1539 (0.9%) | 694 (0.4%) | 1055 (0.6%) | 770 (0.5%) | 760  (0.5%) | 855  (0.5%) | 850  (0.5%) | 820  (0.5%) | 0.611 |
| Other | 5491 (3.0%) | 5188 (2.7%) | 5677 (3.5%) | 6906 (3.8%) | 7840  (4.5%) | 5196 (3.1%) | 7019 (4.2%) | 6340 (3.8%) | 5680 (3.5%) | 5945 (3.7%) | 5455 (3.3%) | 5610 (3.3%) | 6030 (3.6%) | 0.545 |
| **Income** |  |  |  |  |  |  |  |  |  |  |  |  |  |  |
| Low | 72785 (28.1%) | 69592 (26.6%) | 63131 (28.4%) | 62281 (28.1%) | 56798 (27.6%) | 54104 (28.3%) | 51692 (27.9%) | 53380 (30.2%) | 49210 (28.6%) | 46570 (27.6%) | 50640 (29.6%) | 50095 (28.8%) | 49310 (28.5%) | 0.091 |
| Low-Mid | 70898 (27.4%) | 72651 (27.7%) | 58150 (26.2%) | 66379 (29.9%) | 58152 (28.3%) | 51481 (26.9%) | 46977 (25.4%) | 46565 (26.4%) | 48050 (28.0%) | 49580 (29.4%) | 44080 (25.7%) | 47500 (27.3%) | 48600 (28.1%) | 0.901 |
| High-Mid | 63563 (24.5%) | 65087 (24.8%) | 52662 (23.7%) | 50984 (23.0%) | 49356 (24.0%) | 46479 (24.3%) | 48653 (26.3%) | 41480 (23.5%) | 41270 (24.0%) | 39885 (23.6%) | 42605 (24.9%) | 42455 (24.4%) | 41005 (23.7%) | 0.933 |
| High | 51806 (20.0%) | 54765 (20.9%) | 47971 (21.6%) | 42047 (19.0%) | 41171 (20.0%) | 39150 (20.5%) | 37709 (20.4%) | 35250 (20.0%) | 33245 (19.4%) | 32695 (19.4%) | 33930 (19.8%) | 33780 (19.4%) | 33960 (19.6%) | 0.062 |
| **Expected Primary Payer** |  |  |  |  |  |  |  |  |  |  |  |  |  |  |
| Medicare | 137139 (51.6%) | 131172 (48.8%) | 107762 (47.3%) | 105217 (46.5%) | 95426 (45.4%) | 87431 (44.6%) | 85891 (45.5%) | 82260 (45.6%) | 79575 (45.3%) | 77620 (45.1%) | 79515 (45.5%) | 80330 (45.3%) | 79395 (45.1%) | 0.004 |
| Medicaid | 13999 (5.3%) | 13611 (5.1%) | 11874 (5.2%) | 13143 (5.8%) | 13312 (6.3%) | 14154 (7.2%) | 13464 (7.1%) | 13170 (7.3%) | 12320 (7.0%) | 16495 (9.6%) | 17775 (10.2%) | 18680 (10.5%) | 18785 (10.7%) | <0.001 |
| Private Insurance | 88646 (33.4%) | 94163 (35.1%) | 81505 (35.8%) | 80820 (35.7%) | 74494 (35.4%) | 68717 (35.1%) | 63706 (33.7%) | 53945 (32.9%) | 58550 (33.3%) | 58835 (34.2%) | 59730 (34.2%) | 60215 (34.0%) | 58975 (33.5%) | 0.094 |
| Self Pay | 16712 (6.3%) | 18398 (6.9%) | 16304 (7.2%) | 17631 (7.8%) | 18061 (8.6%) | 17268 (8.8%) | 17485 (9.3%) | 17594 (9.7%) | 16690 (9.5%) | 12680 (7.4%) | 11450 (6.6%) | 11245 (6.3%) | 12025 (6.8%) | 0.96 |
| No charge | 1768 (0.7%) | 1891 (0.7%) | 1621 (0.7%) | 1700 (0.8%) | 1459  (0.7%) | 1221 (0.6%) | 1995 (1.1%) | 1370 (0.8%) | 1915 (1.1%) | 1220 (0.7%) | 1045 (0.6%) | 910 (0.5%) | 920 (0.5%) | 0.48 |
| Other | 7343 (2.8%) | 9293 (3.5%) | 8557 (3.8%) | 7812 (3.5%) | 7547  (3.6%) | 7133 (3.6%) | 6357 (3.4%) | 6730 (3.7%) | 6625 (3.8%) | 5320 (3.1%) | 5275 (3.0%) | 5790 (3.3%) | 5755 (3.3%) | 0.667 |
| **Comorbidities:** |  |  |  |  |  |  |  |  |  |  |  |  |  |  |
| CAD | 185749 (69.8%) | 196140 (72.9%) | 169376 (74.2%) | 175161 (77.2%) | 167860 (79.6%) | 157814 (80.4%) | 154123 (81.3%) | 149090 (82.4%) | 145550 (82.7%) | 143460 (83.2%) | 148115 (84.6%) | 153090 (86.3%) | 151240 (85.8%) | <0.001 |
| Diabetes | 67799 (25.5%) | 69532 (25.9%) | 60410 (26.5%) | 61394 (27.1%) | 58056 (27.5%) | 54710 (27.9%) | 55096 (29.1%) | 53035 (29.3%) | 52635 (29.9%) | 52510 (30.4%) | 54485 (31.1%) | 56405 (31.8%) | 56330 (32.0%) | <0.001 |
| Obesity | 19918 (7.5%) | 20560 (7.6%) | 19716 (8.6%) | 22476 (9.9%) | 23055 (10.9%) | 21764 (11.1%) | 23578 (12.4%) | 24170 (13.4%) | 24920 (14.2%) | 25725 (14.9%) | 27420 (15.7%) | 28340 (16.0%) | 29935 (17.0%) | <0.001 |
| Hypertension | 148288 (55.8%) | 153915 (57.2%) | 134019 (58.7%) | 136687 (60.3%) | 132684 (62.9%) | 124908 (63.6%) | 123850 (65.4%) | 119545 (66.1%) | 118695 (67.4%) | 118105 (68.5%) | 122755 (70.1%) | 123975 (69.9%) | 106715 (60.6%) | 0.003 |
| Smoking | 148288 (55.8%) | 153915 (57.2%) | 134019 (58.7%) | 136687 (60.3%) | 132684 (62.9%) | 124908 (63.6%) | 123850 (65.4%) | 119545 (66.1%) | 118695 (67.4%) | 118105 (68.5%) | 122755 (70.1%) | 123975 (69.9%) | 106715 (60.6%) | <0.001 |
| Dyslipidemia | 148288 (55.8%) | 153915 (57.2%) | 134019 (58.7%) | 136687 (60.3%) | 132684 (62.9%) | 124908 (63.6%) | 123850 (65.4%) | 119545 (66.1%) | 118695 (67.4%) | 118105 (68.5%) | 122755 (70.1%) | 123975 (69.9%) | 106715 (60.6%) | <0.001 |
| PVD | 148288 (55.8%) | 153915 (57.2%) | 134019 (58.7%) | 136687 (60.3%) | 132684 (62.9%) | 124908 (63.6%) | 123850 (65.4%) | 119545 (66.1%) | 118695 (67.4%) | 118105 (68.5%) | 122755 (70.1%) | 123975 (69.9%) | 106715 (60.6%) | 0.031 |
| Acute renal failure | 17218 (6.5%) | 24966 (9.3%) | 22684 (9.9%) | 22606 (10.0%) | 21506 (10.2%) | 19829 (10.1%) | 20390 (10.8%) | 19070 (10.5%) | 18950 (10.8%) | 19455 (11.3%) | 20015 (11.4%) | 22000 (12.4%) | 21725 (12.3%) | <0.001 |
| **Hospital Bedsize** |  |  |  |  |  |  |  |  |  |  |  |  |  |  |
| Small | 4534 (8.3%) | 7143 (13.0%) | 5039 (10.9%) | 5022 (10.9%) | 3646 (8.9%) | 4227 (10.9%) | 3640 (9.3%) | 3708 (10.2%) | 3469 (9.9%) | 4837 (14.0%) | 4765 (13.6%) | 4861 (13.7%) | 5464 (15.5%) | 0.017 |
| Medium | 12615 (23.2%) | 13083 (23.7%) | 10476 (22.7%) | 10169 (22.0%) | 8570 (20.9%) | 7552 (19.5%) | 8762 (22.4%) | 8808 (24.3%) | 8720 (24.8%) | 10068 (29.2%) | 10472 (29.9%) | 10273 (28.9%) | 10667 (30.3%) | 0.002 |
| Large | 37268 (68.5%) | 34907 (63.3%) | 30618 (66.4%) | 30993 (67.1%) | 28787 (70.2%) | 26949 (69.6%) | 26771 (68.3%) | 23676 (65.4%) | 23014 (65.4%) | 19600 (56.8%) | 19785 (56.5%) | 20352 (57.4%) | 19116 (54.2%) | 0.002 |
| **Hospital Location** |  |  |  |  |  |  |  |  |  |  |  |  |  |  |
| Rural | 7180 (13.2%) | 6170 (11.2%) | 5970 (12.9%) | 5683 (12.3%) | 4416 (10.8%) | 4808 (12.4%) | 3331 (8.5%) | 3387 (9.4%) | 3264 (9.3%) | 2585 (7.5%) | 2391 (6.8%) | 2332 (6.6%) | 2395 (6.8%) | <0.001 |
| Urban | 47237 (86.8%) | 48963 (88.8%) | 40163 (87.1%) | 40501 (87.7%) | 36587 (98.2%) | 33920 (87.6%) | 35842 (91.5%) | 32805 (90.6%) | 31939 (90.7%) | 31920 (92.5%) | 32631 (93.2%) | 33154 (93.4%) | 32852 (93.2%) | <0.001 |
| **Hospital Region** |  |  |  |  |  |  |  |  |  |  |  |  |  |  |
| Northeast | 9538 (17.5%) | 8334 (15.1%) | 7546 (16.3%) | 7680 (16.6%) | 7354 (17.6%) | 7113 (18.1%) | 6717 (16.9%) | 6256 (17.3%) | 6177 (17.5%) | 5946 (17.2%) | 6081 (17.4%) | 6022 (17.0%) | 6050 (17.2%) | 0.212 |
| Midwest | 13241 (24.3%) | 13107 (23.7%) | 11574 (25.1%) | 10961 (23.7%) | 9720 (23.2%) | 10047 (25.5%) | 9542 (24.1%) | 8317 (23.0%) | 8216 (23.3%) | 8076 (23.4%) | 8172 (23.3%) | 8212 (23.1%) | 7876 (22.3%) | 0.023 |
| South | 22226 (40.8%) | 23852 (43.2%) | 18139 (39.3%) | 19659 (42.5%) | 17329 (41.4%) | 14136 (35.9%) | 15825 (39.9%) | 14715 (40.7%) | 14322 (40.7%) | 14049 (40.7%) | 14259 (40.7%) | 14548 (41.0%) | 14257 (40.4%) | 0.087 |
| West | 9412 (17.3%) | 9936 (18.0%) | 8917 (19.3%) | 7909 (17.1%) | 7485 (17.9%) | 8030 (20.4%) | 7583 (19.1%) | 6904 (19.1%) | 6488 (18.4%) | 6434 (18.6%) | 6510 (18.6%) | 6704 (18.9%) | 7064 (20.0%) | 0.087 |
|  |  |  |  |  |  |  |  |  |  |  |  |  |  |  |
| **LoS** | 3 (4) | 3 (4) | 3 (3) | 3 (3) | 3 (3) | 3 (3) | 3 (3) | 3 (3) | 3 (3) | 3 (2) | 3 (2) | 3 (2) | 2 (2) | 0.111 |
| **Outcomes** |  |  |  |  |  |  |  |  |  |  |  |  |  |  |
| Mortality | 25610 (9.6%) | 23485 (8.7%) | 20814 (9.1%) | 20483 (9.0%) | 18149 (8.6%) | 16164 (8.2%) | 16101 (8.5%) | 14940 (8.3%) | 14235 (8.1%) | 14210 (8.2%) | 13835 (7.9%) | 14270 (8.0%) | 13915 (7.9%) | <0.001 |
| Ventricular Tachycardia | 20639 (7.8%) | 21414 (8.0%) | 18179 (8.0%) | 19473 (8.6%) | 17574 (8.3%) | 17789 (9.1%) | 17139 (9.0%) | 17185 (9.5%) | 16925 (9.6%) | 18210 (10.6%) | 18885 (10.8%) | 19115 (10.8%) | 20435 (11.6%) | <0.001 |
| Ventricular Fibrillation | 11370 (4.3%) | 12276 (4.6%) | 11484 (5.0%) | 12068 (5.3%) | 12233 (5.8%) | 11849 (6.0%) | 12145 (6.4%) | 12085 (6.7%) | 12265 (7.0%) | 12465 (7.2%) | 13380 (7.6%) | 13885 (7.8%) | 14095 (8.0%) | <0.001 |
| Atrial Fibrillation | 36112 (13.6%) | 35654 (13.3%) | 28983 (12.7%) | 26530 (12.2%) | 25778 (12.2%) | 23251 (11.8%) | 24846 (13.1%) | 23160 (12.8%) | 22330 (12.7%) | 23155 (13.4%) | 23915 (13.7%) | 24405 (13.8%) | 24480 (13.9%) | 0.133 |
| Heart Failure | 68597 (25.8%) | 64881 (24.1%) | 53529 (23.5%) | 49961 (22.0%) | 45921 (21.8%) | 41670 (21.2%) | 42372 (22.4%) | 38525 (21.3%) | 37970 (21.6%) | 37660 (21.8%) | 39505 (22.6%) | 41480 (23.4%) | 43895 (24.9%) | 0.516 |
| Ischemic Stroke | 3634 (1.4%) | 3780 (1.4%) | 2969 (1.3%) | 3027 (1.3%) | 2553 (1.2%) | 2509 (1.3%) | 2299 (1.2%) | 2255 (1.2%) | 2125 (1.2%) | 2310 (1.3%) | 2130 (1.2%) | 2270 (1.3%) | 2450 (1.4%) | 0.377 |
| Acute Renal Failure | 18369 (6.9%) | 19465 (7.2%) | 18605 (8.2%) | 21888 (9.7%) | 22510 (10.7%) | 21414 (10.9%) | 21561 (11.4%) | 21765 (12.0%) | 21725 (12.3%) | 23880 (13.8%) | 25020 (14.3%) | 28140 (15.9%) | 28935 (16.4%) | <0.001 |
| Cardiogenic Shock | 18598 (7.0%) | 19840 (7.4%) | 18782 (8.2%) | 20388 (9.0%) | 20647 (9.8%) | 19731 (10.0%) | 20906 (11.0%) | 19785 (10.9%) | 19710 (11.2%) | 21335 (12.4%) | 22055 (12.6%) | 22875 (12.9%) | 23190 (13.2%) | <0.001 |
| **Interventions** |  |  |  |  |  |  |  |  |  |  |  |  |  |  |
| PCI | 133802 (50.3%) | 150198 (55.8%) | 133633 (58.6%) | 142974 (63.0%) | 140506 (66.6%) | 136310 (69.4%) | 134947 (71.2%) | 133620 (73.8%) | 132315 (75.2%) | 132200 (76.6%) | 136000 (77.7%) | 141750 (79.9%) | 143400 (81.4%) | <0.001 |
| Thrombolysis | 8732 (3.3%) | 7814 (2.9%) | 5447 (2.4%) | 5121 (2.3%) | 4734 (2.2%) | 3532 (1.8%) | 3685 (1.9%) | 3475 (1.9%) | 3465 (2.0%) | 3605 (2.1%) | 3045 (1.7%) | 2765 (1.6%) | 2395 (1.4%) | <0.001 |
| CABG | 23184 (8.7%) | 24323 (9.0%) | 19251 (8.4%) | 17984 (7.9%) | 16851 (8.0%) | 13517 (6.9%) | 13015 (6.9%) | 11555 (6.4%) | 10945 (6.2%) | 10355 (6.0%) | 10370 (5.9%) | 9935 (5.6%) | 9175 (5.2%) | <0.001 |

CABG= Coronary Artery Bypass Graft, CAD= coronary artery disease, LoS= length of stay, PCI= Percutaneous Coronary Intervention, PVD= peripheral vascular disease.
